# Towards a promising systematic approach to the synthesis of CZTS solar cells

**DOI:** 10.1038/s41598-023-42641-w

**Published:** 2023-09-18

**Authors:** A. S. Najm, Azza Al-Ghamdi, Majdi T. Amin, Ahmed Al Ghamdi, Hazim Moria, Araa Mebdir Holi, Azher M. Abed, Asla Abdullah AL-Zahrani, K. Sopian, Badariah Bais, Abbas J. Sultan

**Affiliations:** 1 Department of Electrical, Electronics and System, FKAB, Universiti Kebangsaan Malaysia (UKM), 43600 Bangi, Selangor Malaysia; 2 Petroleum Research and Development Center, Ministry of Oil, Baghdad, Iraq; 3 Department of Chemical Engineering, University of Technology, Baghdad, Iraq; 4 Department of Chemistry, College of Science, Imam Abdulrahman Bin Faisal University, P.O. Box 1982, 31441 Dammam, Saudi Arabia; 5 Basic & Applied Scientific Research Center (BASRC), Renewable and Sustainable Energy Unit, Imam Abdulrahman Bin Faisal University, P.O. Box 1982, 31441 Dammam, Saudi Arabia; 6 Department of Mechanical Engineering Technology, Yanbu Industrial College, 41912 Yanbu Al-Sinaiyah City, Saudi Arabia; 7 Department of Chemical Engineering Technology, Yanbu Industrial College, 41912 Yanbu Al-Sinaiyah City, Saudi Arabia; 8 Department of Physics, College of Education, University of Al-Qadisiyah, Al-Diwaniyah, Al-Qadisiyah 58002 Iraq; 9 Department of Air Conditioning and Refrigeration, Al-Mustaqbal University, Babylon, Iraq; 10 Imam Abdulrahman-Bin Fiasal University, Eastern Region, Dammam, Saudi Arabia; 11 Department of Mechanical Engineering, Universiti Teknologi PETRONAS, 32610 Seri Iskandar, Perak Darul Ridzuan, Malaysia

**Keywords:** Energy science and technology, Nanoscience and technology

## Abstract

This study aims to enhance the CZTS device's overall efficiency, the key research area has been identified in this study is to explore the effects of a novel, low-cost, and simplified, deposition method to improve the optoelectronic properties of the buffer layer in the fabrication of CZTS thin film solar cells. Herein, an effective way of addressing this challenge is through adjusting the absorbers' structure by the concept of doping, sensitized CdS thin film by the bi-functional linker, and an environmentally friendly catalytic green agent. The Linker Assisted and Chemical Bath Deposition (LA-CBD) method was introduced as an innovative and effective hybrid sensitization approach. In the one-step synthesis process, Salvia dye, Ag, and 3-Mercaptopropionic acid (MPA) were used. Generally, the results for all samples displayed varying bandgap as achieved between (2.21–2.46) eV, hexagonal structure with considerably decreased strain level, broader grain size, and dramatically enhanced crystalline property. Hence, the rudimentary CdS/CZTS solar cell devices were fabricated for the application of these novel CdS films. Preliminary CZTS thin film solar cell fabrication results in the highest conversion efficiency of 0.266% obtained CdS + Salvia dye, indicating the potential use of the CdS films as a buffer layer for CZTS photovoltaic devices.

## Introduction

Considering the growing demand for energy for both domestic and commercial use, there is needed to provide an affordable renewable source of energy to sustain economic growth^1,2^. Nowadays, attention is tending to renewable, sustainable energy to reduce energy consumption and environmental pollution^3^. Recently, there has been a growing interest in the uses of nanomaterials based on transition metal chalcogenides, namely those belonging to the II-VI semiconductor group. This interest comes from the remarkable physical characteristics shown by these materials^4,5^.

The thin-film semiconductor has a smooth layer of micro to nanometers depending on the nucleation processes and the growth mechanism. In our previous work, we discussed the mechanism of synthesis of CdS thin film as a buffer layer, also we clarify how the deposition conditions and thickness have a strong impact on the chemical and physical characteristics of the films^6,7^. Thus, it should be determined the crystallographic and microstructural features of CdS film in order to enhance both the electrical and optical properties, that depends on several parameters, including growth kinetics, the source of impinged fine particles, the chemical nature of the source, and substrate-surface topography^8^. Therefore, it is essential to know how specific issues affect the structure during film growth.

In this work, the way has been open for improving the physical and chemical properties of CdS thin films through applying annealing side by side with the principle of doping and sensitization. Buffer layer deposition by chemical bath process is a mature research area, thus, it is not surprising that numerous variants of CBD recipes for the deposition of different types of semiconducting materials can be found in published literature. Having a standard recipe for CdS deposition that is reproducible is advantageous for a more accurate comparison of results, both in this study and in future investigations. Consequently, the use of appropriate growth procedures allows for the simultaneous achievement of a wide band-gap window (2.4 eV) and enhanced electrical conductivity^9^. The procedure includes immersing the substrate in a solution including the precursors, wherein the thin film is acquired by the regulated precipitation of the compounds from the solution^10^.

In principle, CBD demands that the product of the metal ion and chalcogenide concentrations must be higher than the solubility of the desired product. Nevertheless, the total thickness achievable is limited by the availability of reactants in the solution. The monitoring of film thickness, composition, and density are collectively governed by various parameters which affect the solution chemistry including solution composition, duration of deposition, pH, temperature, and chemical nature of the substrate. Chemical preparation of doping occurs during the growth phase, and it may be achieved in situ by introducing controlled quantities of a salt solution containing the doping atom into the reaction solution. This method allows for doping without causing any significant harm to the lattice structure. The most successful approach to address the need for post-deposition treatments is by in-situ doping using elements such as aluminium, indium, silver, boron, and gallium^11–14^. Indeed, owing to their widely scattered state, metal nanoparticles in solution have a tend to spontaneously agglomerate and coagulate, thus requiring maintenance to prevent such occurrences^15^. To reduce this effect bi-functional bridging ligands have been incorporated during the reaction to form a more rigid, thus obstructing the growth to limiting agglomeration^16^. So far, thiols are the best ligands to monitor the nucleation and growth of II–VI semiconductor nanocrystals. Moreover, more stable CdS nanoparticles could be prepared solely employing mercaptopropionic acid or cysteamine without polymeric stabilizers due to the tight interaction between Cd ions and thiols.

**Figure 1:**
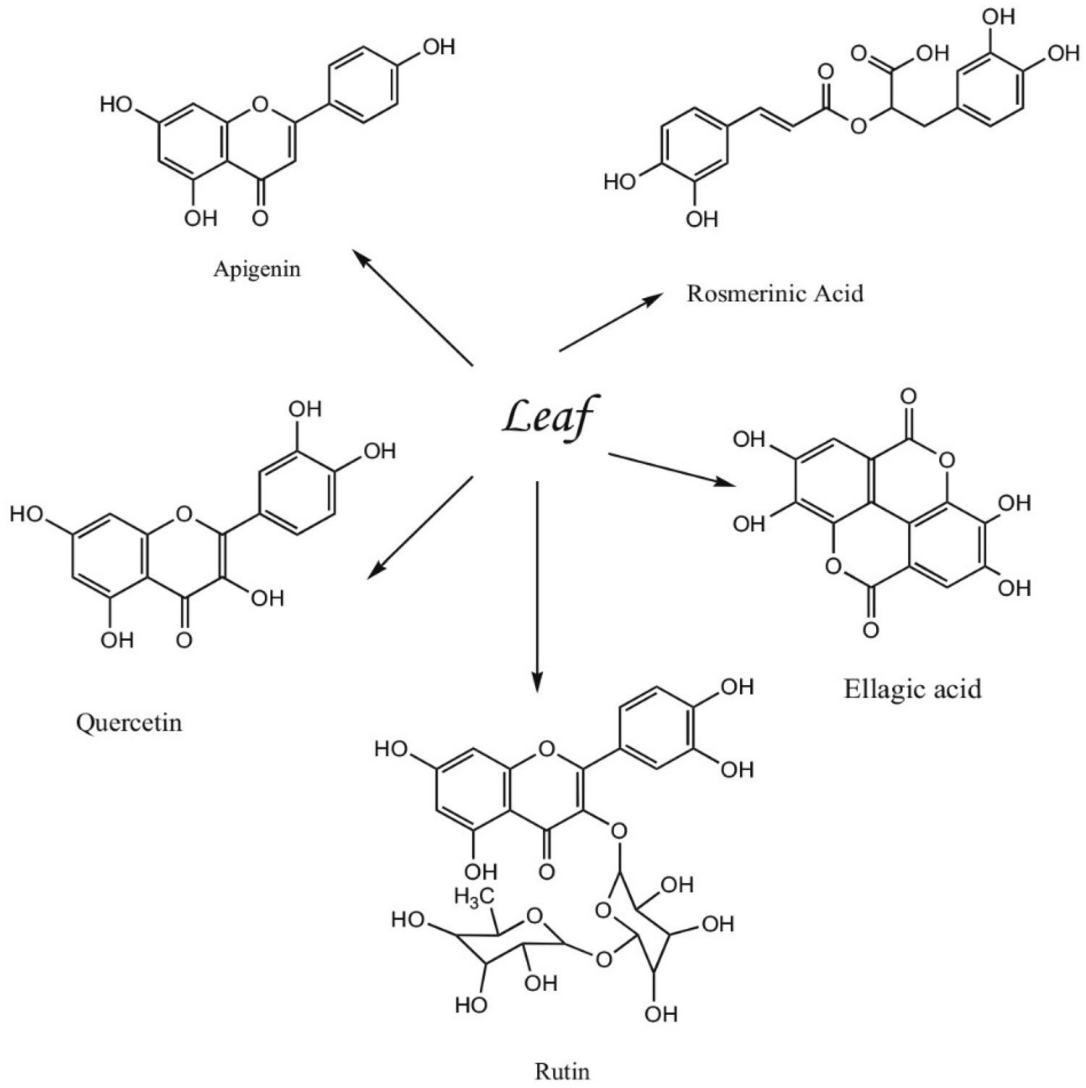
*Salvia officinalis* L. leaf composition^18^.

On the other side, as an alternative capping agent, Salvia has been identified as a potential capping agent due to its high content of phenolic compounds, including caffeic acid, vanillic acid, ferulic acid, luteolin, apigenin, quercetin, rosmarinic acid, and their derivatives (Fig. 1). This alternative capping agent has been utilized in the preparation of nanoparticles to reduce the presence of metal salts, without the need for additional reducing or stabilizing agents. The presence of polyphenol in ethanol extracts such as salvianolic acid, rosemarinic acid, and luteiolin gluci side serves as reducing and stabilizing agents for nanoparticles of gold salts^17^.

In summary, several experiments on the properties of nanostructures alter the size of nanoparticles through various methods such as adjusting the ratio of the precursors, varying reaction conditions, varying capping agents, etc. To date, very rare works have been conducted on the effect of nanoparticles composition and the effect of capping coordination on CdS nanoparticles properties capped by an organic molecule. Therefore, the objective of the current study is to acquire a CdS thin film with a specific capping agent by solution processing methods, without the need for further modifications after deposition. An effective approach to address this issue is through adjusting the absorbers ' structure by the concept of doping, sensitized CdS thin film by the bi-functional linker, and an environmentally friendly catalytic green agent. It has to be asserted that the scope of this objective is not to produce very high-efficiency CZTS PV devices because the research in this field at the thin film laboratory at the National University of Malaysia (UKM) is still very much in its infancy stages (compared to nearly four decades of experience in world-class research laboratories). Therefore, the investigation is aimed at fabricating functioning CZTS devices instead. The results obtained from this section can then be used as a platform upon which future research in this area can be developed.

## Experimental details

### LACBD process

CZTS solar cells have been made with soda-lime glass (SLG) as the substrate material. The SLG material is sectioned into 1 cm × 1 cm sections for characterization purposes, and 1.25 cm × 2.5 cm parts for the fabrication of CZTS solar cell devices. RF sputtering was utilized to deposit thin films of Mo, CZTS, i-ZnO, ITO, and Al. A two-step approach was employed in the assembly of the absorber layer, which involved the deposition of stacked metallic precursor via sputtering, followed by a sulphurization process. A ceramic target of single quaternary CZTS with a diameter of 2 inches and a purity of 99.99%, sourced from Applied Science Corp in Korea, was utilized. The composition of the target comprised 22% Cu, 15% Zn, 13% Sn, and 50% S. The RF-sputtering technique was employed to deposit the target onto pre-cleaned SLG substrates at a temperature of 180 °C. The sputtering deposition chamber's base pressure was reduced to 0.1 mTorr using a turbomolecular pump. Working pressure of 7.5 mTorr was maintained for all deposition runs by introducing 4 sccm of high-purity Ar (Linde, 99.9999%) as the working gas. The films that underwent sputtering were subjected to sulphurization in a vacuum tube furnace at a temperature of 560 °C for 1 h, with a ramp rate of 5 °C/min, 30 mg of sulphur (S) powder, and 0.5 atm of nitrogen background pressure.

**Figure 2:**
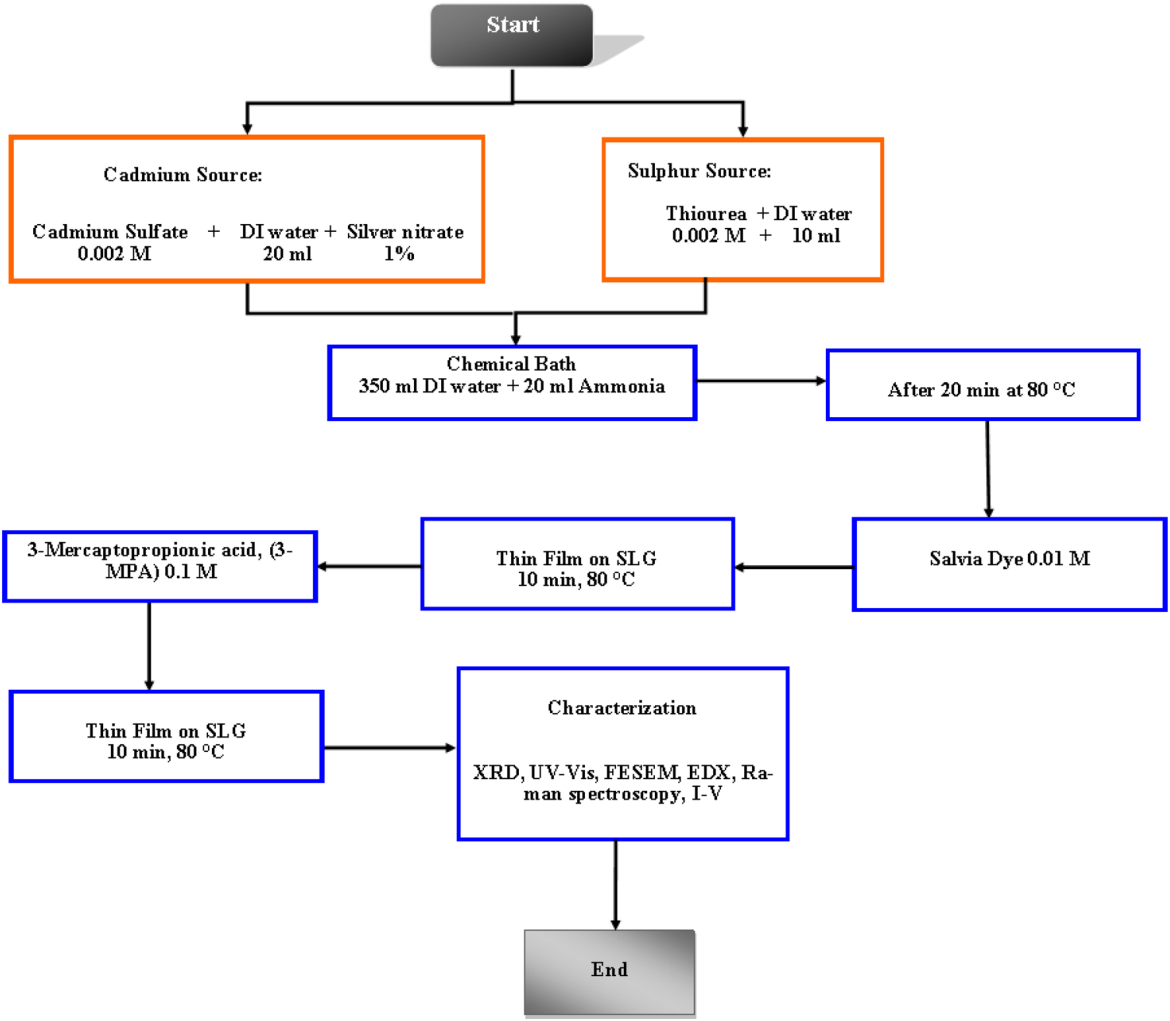
Synthesis of CdS thin film (mix all).

Then after, CdS thin film has been synthesised using a modified method based on our previous work^7^. Initially, we prepared the fresh Sage Plant (from an Arabic store in Malaysia), then we prepared the silver nitrate (AgNO_3_) stock solution, and last, we prepared the MPA stock solution. The pH level has been adjusted to 10 using KOH base^19^. This method used two experimental sessions. The first stage involved the synthesis of CdS using three different methods: Basic CdS thin film alone, CdS mix with one of each stock, and mixing between each stock. In the second stage, CdS was optimized using a hybrid process that included simultaneous mixing as; Salvia dye + AgNO_3_, Salvia dye + MPA, and Salvia dye + AgNO_3_ + MPA. The synthesis of CdS thin film is depicted in Fig. 2, specifically in the mix-all case. For optimising, the annealing has been utilized at 250 °C for 10 min, then send to characterization to select the optimized one for fabrication.

**Figure 3:**
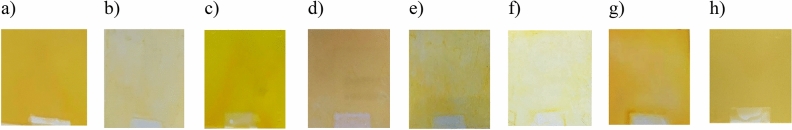
CdS thin films deposited; at: (**a**) Basic CdS, (**b**) CdS + Ag, (**c**) CdS + MPA, (**d**) CdS + Salvia dye, (**e**) CdS + Ag + MPA, (**f**) CdS/Ag/Salvia dye, (**g**) CdS + MPA + Salvia dye, (**h**) CdS + Ag + MPA + Salvia dye.

The considerable absorption of visible light by these thin films offers an intuitive understanding of high CdS loading, which is also shown by the strong colouring of the electrodes in Fig. 3's inset. From this point on and throughout the remainder of the work, we will be denoted using the terminology outlined in Fig. 3.

Following is the buffer layer deposition, CdS, i-ZnO, and ITO are placed as the subsequent layers. The i-ZnO layer serves as a further buffer layer above the buffer layer. ITO is a layer of transparent conducting oxide that gathers photo-generated electrons before their transfer to the Al-top metal grid. RF sputtering is used to deposit both the i-ZnO and ITO layers. The assessed thickness of the i-ZnO layer is between 80 and 100 nm, whereas the estimated thickness of the n-ZnO layer is between 700 and 800 nm. Moreover, RF sputtering is used to deposit the front grid of aluminium metal.

### Characterisation

The optical characteristics were assessed throughout a spectral range spanning from 350 to 650 nm, using a Lambda 950 UV/VIS/NIR spectrophotometer supplied by Perkin-Elmer, USA. The films' structural characterizations were evaluated at room temperature using an AXS-D8 Advance Cu-Kα diffractometer provided by Bruker Corp, USA. The X-ray diffraction (XRD) patterns were examined in a 2Θ range, with a step size of 0.02°, covering from 10° to 80°. The analysis was conducted using Cu-Kα radiation wavelength (λ) of 1.5408 Å. The scanning electron microscope (SEM) model FEI Quanta 400F, which employs field emission technology, has been enhanced with the addition of an Oxford-Instruments INCA 400 X-Max detector. This modification enables the SEM to perform energy-dispersive x-ray spectroscopy (EDX) observations. The device has the capability to attain a magnification level of 300x, accompanied by a spot size of 1 mm × 1 mm. Additionally, it functions at an accelerating voltage of 20 kV. The Raman spectra of the films were obtained using a Renishaw InVia Raman Microscope equipped with a charge-coupled device detector and a grating with a density of 2400 lines per mm. The experimental setup included the use of an argon-ion laser, which operated within a certain range of excitation wavelength and power. The excitation wavelength was set at 514 nm, while the power ranged from 10 to 50 mW. The light I–V properties of solar cells were used to assess their photovoltaic qualities. The experimentation was carried out with a AAA class SSPN-X150T solar simulator, provided by Light Doctor Optical Corporation in Taiwan, which emitted AM 1.5 G irradiation. The IVDN-250E source meter was used for this purpose.

## Results and discussions

### Optical analysis

**Figure 4:**
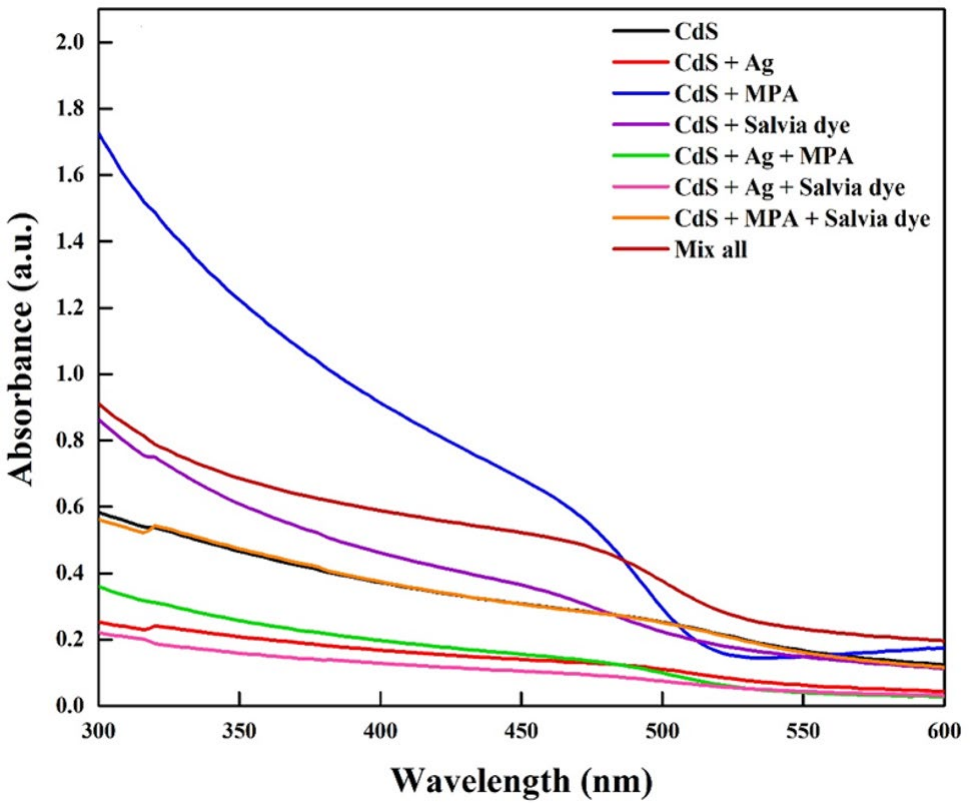
Systematic study for CdS with and without additives.

The optical characteristics of solar cells are crucial in assessing their efficiency^20^. The optical absorbance spectra of CdS thin films in all of the cases were shown in Fig. 4.

The distinct absorption spectrum with a sharp onset and peak suggests a narrow size distribution. These characteristics are not achievable by directly growing a thin layer of CdS^8^. Obviously, the optical properties are influenced by deposition techniques^21–23^. As compared with our previous work^6,7^, samples under annealing treatments also have lower bandgap energy than those under the same conditions. This pattern should be compared with the sample structural properties to find the bandgap shifting origin. It may be attributed to film thickness, grain size, or various phases^24^.

Since the absorbance is directly related to the amount of CdS sensitizer uploaded, it can find that the high loading amount of CdS as-deposited was very clear in (CdS + MPA) sample, whereas (CdS + Ag + Salvia dye) sample exhibited the lowest absorption spectra. Bonding of the MPA molecule to the metal centres on the CdS surface injects electron density, which is represented in the valence band by the proper energy levels^25^. The observed absorption edge exhibited a little blue shift, which could be due to a decrease in electron density inside the valence band. This phenomenon is likely a result of the combined effects of all additives, as shown by the peak intensity. The combination of MPA with Salvia dye exhibits considerable interactions between the adsorbed dye and MPA molecules, which therefore contributes to the observed widening of the absorption spectrum profile^26^. The addition of Salvia dye to MPA resulted in a decrease in absorbance in comparison to MPA alone. The present study's results align with the findings of Hassan et al., which indicate that the absorption intensity is reduced due to competition between the two chemicals present on the surface^27^.

The Tauc's plot method is used for calculating the optical bandgap values. This method involves analyzing the relationship between dispersion and the basic absorption edge, which is the straight bandgap of the semiconductor^28^. The direct semiconductor exhibits a correlation between its optical bandgap and optical absorption coefficient (α) during the transition process:

1 $$\alpha hv={B\left(hv-{E}_{g}\right)}^{n}$$

**Figure 5:**
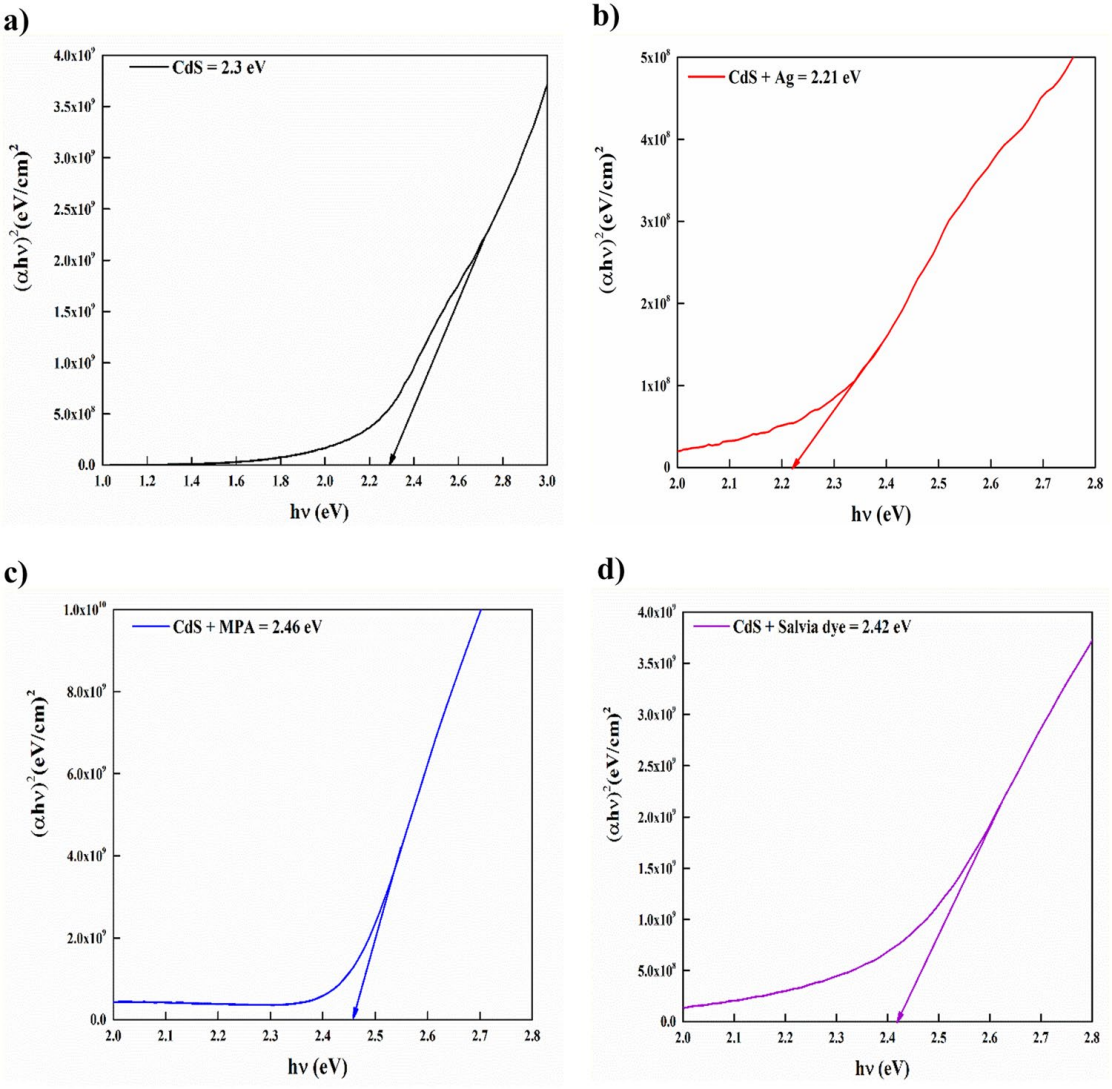

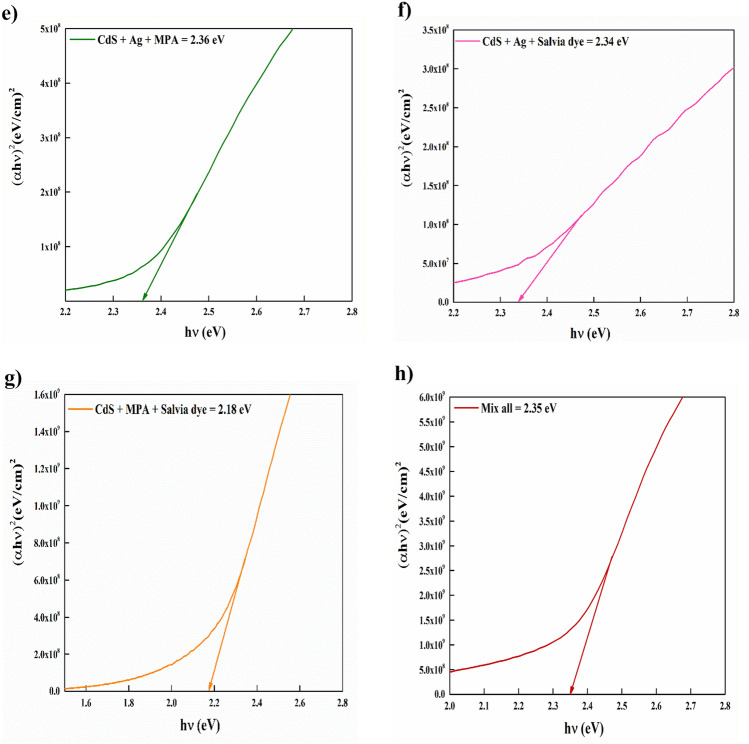
Variant of (*αhν*)^2^ with photon energy (*hν*) for CdS with and without additives at: (**a**) Basic CdS, (**b**) CdS + Ag, (**c**) CdS + MPA, (**d**) CdS + Salvia dye, (**e**) CdS + Ag + MPA, (**f**) CdS/Ag/Salvia dye, (**g**) CdS + MPA + Salvia dye, (**h**) CdS + Ag + MPA + Salvia dye.

The previous formula involves the variables α, $hv$, Eg, *B*, and n, where α represents the coefficient of absorption,$hv$ denotes the photon energy, Eg signifies the direct bandgap energy, *B* stands a comparative constant, and n = ½, is the assumed value for the direct bandgap nature of the material, depends on the type of transition. The average band gap was estimated from the intercept of linear a portion of the ($\alpha$ hν)^2^ vs. hν plots on hν axis as shown in Fig. 5.

Both (CdS + MPA) and (CdS + Salvia dye) showed a widening bandgap, which could be assigned to the Moss–Burstein effect^29^, due to increasing the hopping of charge carriers. In the instances of CdS + Ag + Salvia dye and CdS + MPA + Salvia dye films, the coexistence of these doping agents and an important absence of sulphur would result in heightened donor levels within the CdS bandgap. The reduction of bandgap can be related to the phenomenon wherein the conduction band of CdS is permitted to expand into the forbidden region as a result of the merging of donor levels with the conduction band during doping^30^. For other samples, the reduction of the energy bandgap is assumed to be caused by donor-level degeneracy and disorder-induced band tailings. This is because almost all of the material's atoms are already formed, and thermal vibrations may have moved the atoms out of their original positions, causing them to collide with each other. These defects may serve as trap centres and impact the energy bandgap^31^. The E_g_ values obtained to follow the order in Table 1.

**Table 1:** Bandgap vs urbach energy.

Samples	Bandgap (eV)	Urbach energy (eV)
CdS	2.30	0.52
CdS + Ag	2.21	0.39
CdS + MPA	2.46	0.14
CdS + salvia dye	2.42	0.45
CdS + Ag + MPA	2.36	0.22
CdS + Ag + salvia dye	2.34	0.39
CdS + MPA + salvia dye	2.18	0.49
Mix all	2.35	0.39

**Figure 6:**
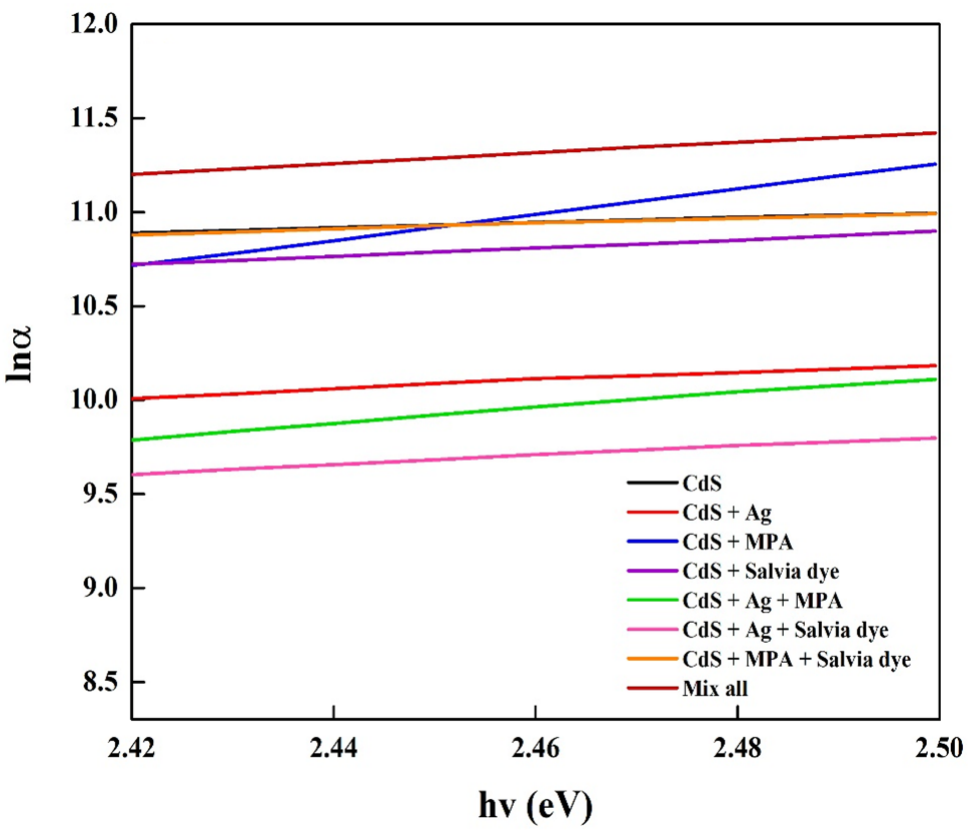
Urbach energy of the CdS deposited for different CdS additives at: (a) Basic CdS, (**b**) CdS + Ag, (**c**) CdS + MPA, (**d**) CdS + Salvia dye, (**e**) CdS + Ag + MPA, (**f**) CdS/Ag/Salvia dye, (**g**) CdS + MPA + Salvia dye, (**h**) CdS + Ag + MPA + Salvia dye.

Whereas, when photon energies decline lowers the bandgap energy, the absorption decreases more gradually than the energy dependence calculated from higher energies. The phenomenon of expanding sub-band absorption may be seen as analogous to the absorption observed in a highly doped crystalline semiconductor. This absorption behaviour is characterized by the Urbach Law, which describes the typical distribution or width of the band tail states^32^, Fig. 6.

In our cases, the change in Urbach's energy suggesting high film disorder may be due to the surface roughness induced by doping. Indeed, there is an inverse relationship between both Urbach energy and bandgap, so it is observed that the E_g_ decreases as the E_u_ value rises. The above-mentioned interpretation of the rise in bandgap energy with absorption intensity confirms this result; that is to suggest, the higher intensity results in films with more order and lower density of localized states. This correlation involving bandgap energy and Urbach energy is comparable to what Melsheimer and Ziegler reported for thin films of tin dioxide, where the band tail reduces from amorphous to polycrystalline structure^33^. Urbach energy decreases as such passes from thinner to thicker films, signifying a transition from disorder to order.

### Structural properties

**Figure 7:**
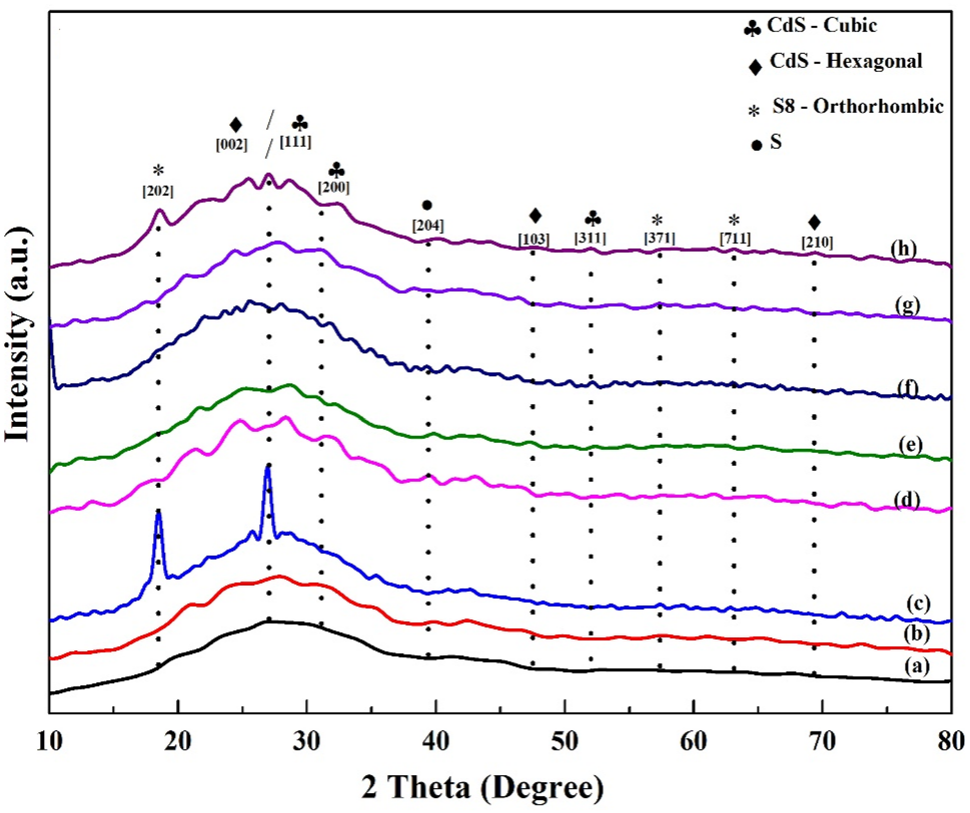
X-ray diffraction patterns for different CdS additives at: (**a**) Basic CdS, (**b**) CdS + Ag, (**c**) CdS + MPA, (**d**) CdS + salvia dye, (**e**) CdS + Ag + MPA, (**f**) CdS/Ag/salvia dye, (**g**) CdS + MPA + salvia dye, (**h**) CdS + Ag + MPA + salvia dye.

The crystal structure of the films was analyzed using X-ray diffraction (XRD) patterns. The phases of CdS were examined, and all of the characteristic XRD patterns are displayed in Fig. 7.

More than a few A prominent diffraction peaks such as 26.7°, 30.6°, and 51.62° indexed to (111), (200), and (311) lattice plans, correspondingly, matching the cubic phase of CdS (JCPDS-89-0440) could be recognized fully in samples, display the steady presence of CdS as the main base in all samples that have examined. Besides, the exact identification of the crystal structure of CdS thin film shows the main strong peak at 26.84°, indexed to (002) with three weaker (103), and (210) peaks of hexagonal CdS planes (JCPDS No. 01-077-2306) has been appearing with diffraction peaks (47.87°, and 69.34°). The polycrystalline hexagonal and cubic configurations of CdS have unpredictable deposition orientations and display many prominent diffraction peaks.

For CdS original pattern Fig. 7a, the main strong peak was around 27.08°, which indicates an increase in crystallinity^34^. Figure 7b illustrates the slight displacement of CdS-doped Ag from their pre-doping positions, at 2θ = 27.84°, oriented along the (002) (JCPDS-00-041-1049). Changes in peak position and FWHM suggest that Ag doping affects the microstructure, crystal quality, and lattice parameters^35^. Figure 7c, showed that the strong peak a little bit shifting positioned at 2θ = 26.94°, is oriented along with the (111) and (002), directions and is in good agreement with the (JCPDS-01-075-0581) and (JCPDS-01-080-0006), suggesting both cubic and hexagonal form. However, the appearance of a sharp peak relative to (202) (JCPDS-01-074-1465) indicates the formation of S_8_-Orthorhombic, since when MPA interacts with cadmium ions most of it forming CdS layers, while a small amount of it which is unreacted, precipitates on the surface of the CdS layer. This is due to, as with annealing at temperature 250 °C, led to stimulates the thiol group in MPA that is not reacted between CdS layers to form S_8_. Figure 7d shows that there is no distinct peak of Salvia dye that shows the synthesis CdS is carried out through reaction without changing in structure. However, the introduction of the dye has had an impact on the shift in position. suggesting that these bands originate from the overlap of multiple peaks and assigned these structures to the hexagonal phase. The peaks (100) and (101) for CdS with Salvia dye, are less broadened, owing to an increase in size^36^.

No peak of elemental silver is present in Fig. 7e, which means there is no simple deposition of elemental Ag. However, from previous work^6^, the same case without annealing showed a new peak at 18.58° has been appearing oriented along the (202), suggesting the new structure related to S_8_-Orthorhombic (JCPDS-01-083-2285). It can explain this in terms of the impact of mixing Ag and MPA in the same reaction, as after Ag^1+^ donated electrons to CdS, it transforms from the oxidative state Ag^1+^ to Ag^2+^, which in turn returns to its initial oxidative state Ag^1+^ by acquiring an electron from the surplus sulphur ions from the MPA. S^−2^ anion work as a supplier of electrons to Ag^2+^. This donation resulted in to form of S neutral and it rapidly reacted with S^−2^ to form the S_8_ orthorhombic. According to the thermodynamics S_8_ formed by melting of MPA has a stronger bond than S_8_ from Ag^+^. So, S_8_ that formed from Ag before annealing was damaged after annealing, due to it is thermally unstable and the S–S bonds in S_8_ are easy to break at 250 °C. The mixing of Ag with Salvia dye was observed in Fig. 7f, which resulted in the shifting of the random structure indicating the increase of crystalline size due to the annealing effect which is matching with the hexagonal phase (JCPDS-01-075-1545). Figure 7g depicts an additional instance of mixing that occurred between MPA and Salvia dye. In comparison to Fig. 7c and e, it is apparent that the mixture is positioned amidst the two, thereby exhibiting efficacious attributes from both and being centrally located.

Figure 7 relates to the sample with the most heterogeneous structure (h). The observation of several peaks indicates the possible existence of polycrystalline films. Nevertheless, the continuous growth of CdS in a specific orientation and the impact of each addition, which were concurrently introduced in the same reaction, yielded peaks that were comparatively less intense in contrast to alternative situations. Despite this, two distinct phases associated to unreacted sulphur were observed. The description can be elucidated by considering the reaction conditions. It is noteworthy that MPA was introduced to the reaction after 20 min in all scenarios, except for this particular case where MPA was added after 30 min. This alteration was made in response to a change in the process, specifically the addition of Salvia dye after 20 min, followed by a 10-min waiting period before stopping the reaction. In this case, earlier CdS has formed two layers, the second layer started after 18 min while MPA was the second source of sulphur adding to the reaction and helped to make a compact, homogenous layer, but when added after 30 min, while approximately most of reacted formed CdS product, here MPA as a second source does not have enough Cd salt to react so it becomes excess without reacted and formed in two phases of sulphur as S (JCPDS-01-074-2108) and S_8_ (JCPDS-01-085-0799). Moreover, the S_8_ was degraded at 250 °C and form a new S_8_ from MPA that was unreacted and deposited inside the layers. Table 2 presents the estimated sizes of crystallites along with their corresponding d-spacing values. The range of observed values, between 25.5 and 62.6 nm, suggests that the composition of polycrystalline CdS films is comprised of particles in the form of nanocrystals.

**Table 2:** Structure characterization of CdS films.

Sample code	Angle 2θ (°)	*hkl* plane	The inter-planer spacing ‘d’ (nm)	FWHM ‘β’ peak width (°)	Crystallite size (nm) ‘D’	Lattice strain (%)
(a) Basic CdS	27.08	(111)/(002)	0.33	0.17	50.4	0.311
(b) CdS doped Ag	27.84	(101)	0.31	0.32	25.5	0.577
(c) CdS capped MPA	26.94	(111)/(002)	0.33	0.3	28	0.545
(d) CdS + salvia dye	24.8, 28.32	(100), (101)	0.35, 0.31	0.13, 0.17	62.6, 50.6	0.273, 0.294
(e) CdS + Ag + MPA	28.73	(111)/(002)	0.31	0.24	35	0.412
(f) CdS + Ag + salvia dye	27.82	(101)	0.32	0.18	46.8	0.322
(g) CdS + salvia dye + MPA	25.35	(002)	0.35	0.3	27.9	0.582
(h) CdS + Ag + salvia dye + MPA	27.1	(002)	0.32	0.3	28	0.543

### Morphological analysis

The porosity of the basic CdS samples reveals that pinhole-free and dense samples cannot be prepared without introducing any treatment. Many uncovered areas of the substrate are seen because there is no uniform distribution of the grains. Obtaining a layer that is free of pinholes is one of the most important requirements for CdS thin films before they can be used as a buffer layer in thin-film solar cells. The CdS doped Ag FESEM micrograph has unregular grain size and less packaging density, which indicated that the grain size and crystal quality declined significantly. This finding may be due to the interdependence of grain size and film thickness, as a result of the process of ion exchange that diffused into CdS films after Ag existence led to thickness gradually decreasing, thus leading to the degradation of crystal quality^37^. And therefore, the EDX spectrum exhibits a high degree of concordance with the experimental outcomes.

**Figure 8:**
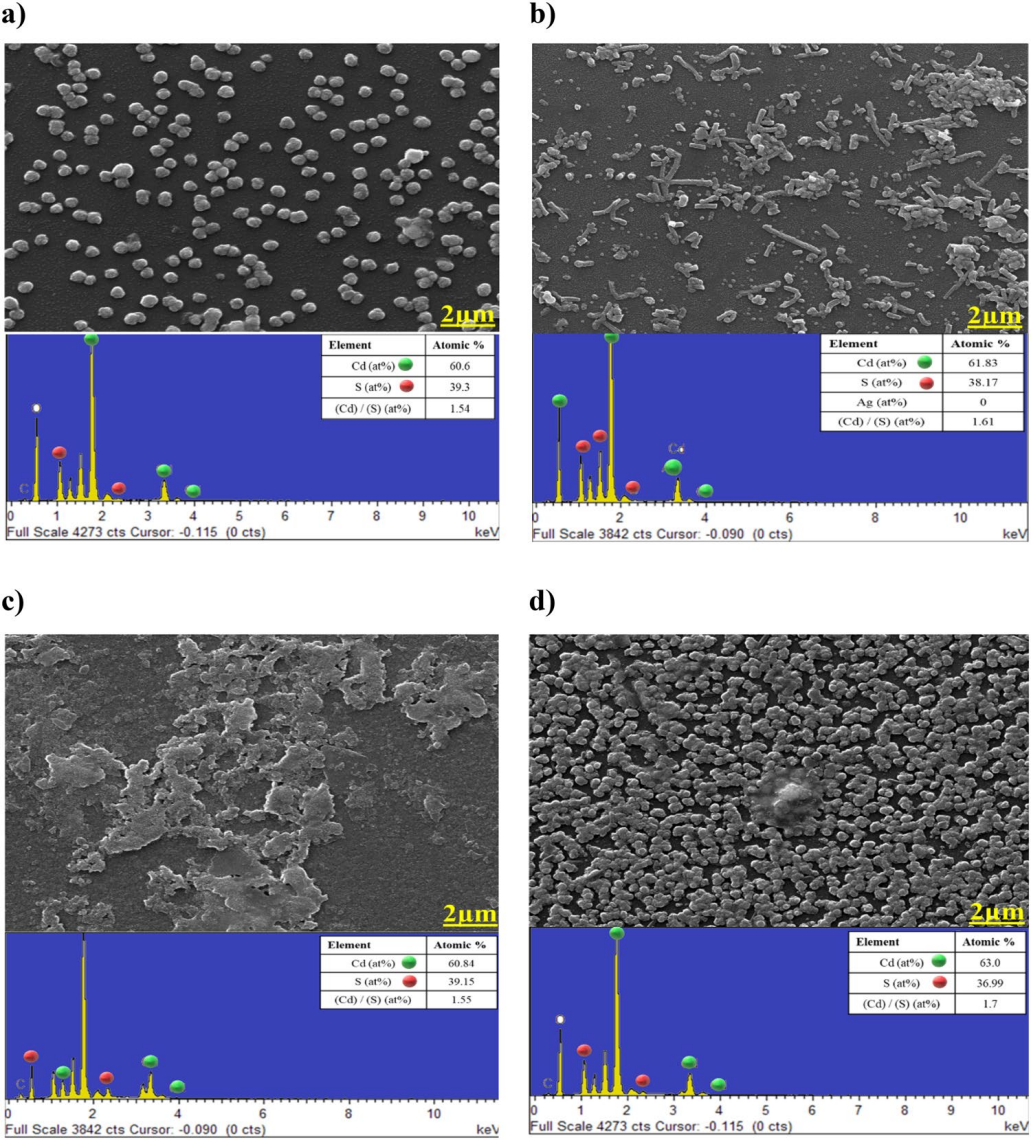
FESEM and EDX for single cases: (**a**) Basic CdS, (**b**) CdS + Ag, (**c**) CdS + MPA, (**d**) CdS + salvia dye.

Samples of (CdS + MPA) simply have dense, compact, and pinhole-free growth on the substrate, Fig. 8c. Dense, compact, and uniform grain distribution that grown after the inclusion of MPA has improved optoelectronic properties in comparison to other samples. The phenomenon can be ascribed to multiple active sites present on the surface, which facilitate the nucleation of CdS. The periodic distribution of larger white spots on the film surface is caused by the presence of adsorbed and self-assembled colloidal particles, which constitute the impurity phase. The films' surface morphologies display a pattern of regularly distributed agglomerated white spots, as well as a limited number of smaller ones^38^. The observed phenomenon is thought to be caused by segregated sulphur atoms that have precipitated at the grain boundaries. In addition, EDX resulted reveal that, even after MPA, the atomic% almost not changed, which refer to the effect of inserting MPA, that give CdS more stability. Figure 8 depicts the presence of spherical nanoparticles without any vacant spaces. The film, on the other hand, is composed of a thin layer of small crystallites, and the nanoparticles are transformed into smaller clusters due to the diffusion of a significant number of CdS nanoparticles. Although, EDX showed that, it is a decline in sulphur atomic %, which is maybe due to the effect of dye as organic material. Even that, it does not impede the homogenous of CdS formation.

**Figure 9:**
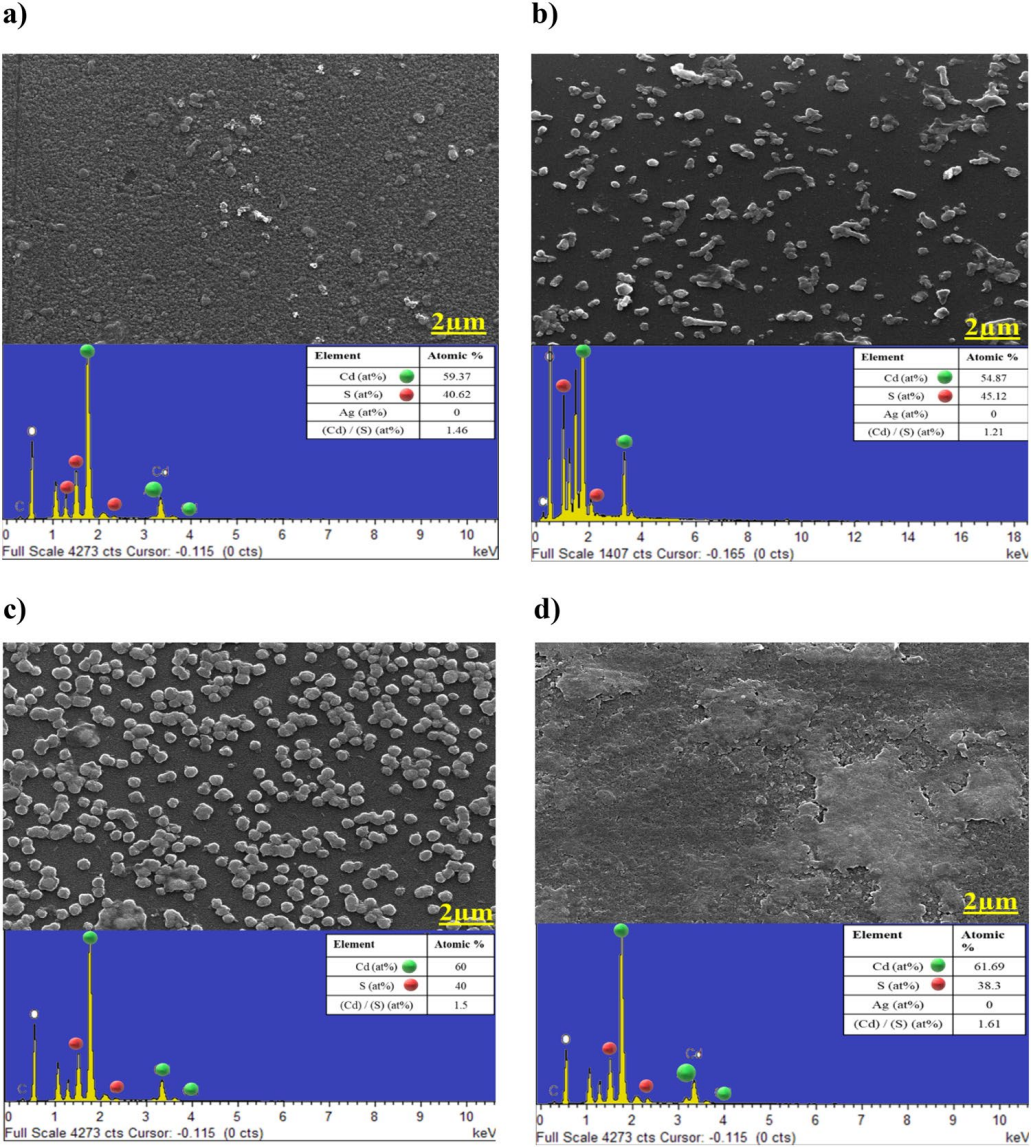
FESEM besides EDX for multiply cases: (**a**) CdS + Ag + MPA, (**b**) CdS + Ag + salvia dye, (**c**) CdS + MPA + salvia dye, (**d**) CdS + Ag + MPA + salvia dye.

Figure 9a illustrates the observation of a non-uniform distribution of particles upon deposition with a mixture of spherical. The structure is changed due to the substitution between MPA and Ag ions. The introduction of Ag into CdS creates vacancies which are subsequently filled by MPA, leading to a denser structural arrangement. The phenomenon can be elucidated as follows: as the concentration of sulphur atoms in the CdS host matrix increases, there is a corresponding decrease in the size of the spherical structure. The reduction in size may be ascribed to the growth limitation of the CdS thin film, which arises from the saturation of sulphur atoms inside the CdS structure^39^. The presence of a distinct diffraction peak in conjunction with the XRD result suggests that a satisfactory quantity of CdS has been deposited. The first stages of film formation are likely attributed to the adsorption of CdS particles onto the substrate, resulting in a uniform growth pattern. The presence of extra sulfur is observable in the crystalline structure of S_8_ Orthorhombic crystals. The induction of stress in the CdS matrix through Ag-doping with Salvia dye is linked to surface defects present in the film. This is evidenced by the observed alteration in the intensity of the XRD pattern. As depicted in Fig. 9b, the external appearance is without any visible surface cracks or imperfections. However, EDX showed that rises in sulphur and dropped in cadmium, due to the effects of both silver and Salvia dye that is obstructing the formation of CdS. Figure 9c depicts the surface morphology of CdS film when MPA and Salvia dye were combined. Small grains and some pinholes are detected, that can cause the device to leakage the current^40^. This can be due to the fact that nanocrystalline agglomerations are small clusters/grains. The alterations in the composition ratio of CdS thin films, as illustrated in Fig. 9d, lead to an adjustment in their morphology from granular structures to more condensed and compact nanoparticles. Several impurities were detected due to contamination on the surface of the film. The present sample exhibits a multi-layered structure. The film exhibits a cluster structure and non-uniform particles that show a unique and clearly defined morphology. The quantity of aggregates increases proportionally with the depth of specific materials present in a solution. The influence of multiple compositions on the reaction is an important variable in the formation of aggregates via secondary nucleation and film growth^41^.

### Raman spectroscopy

**Figure 10:**
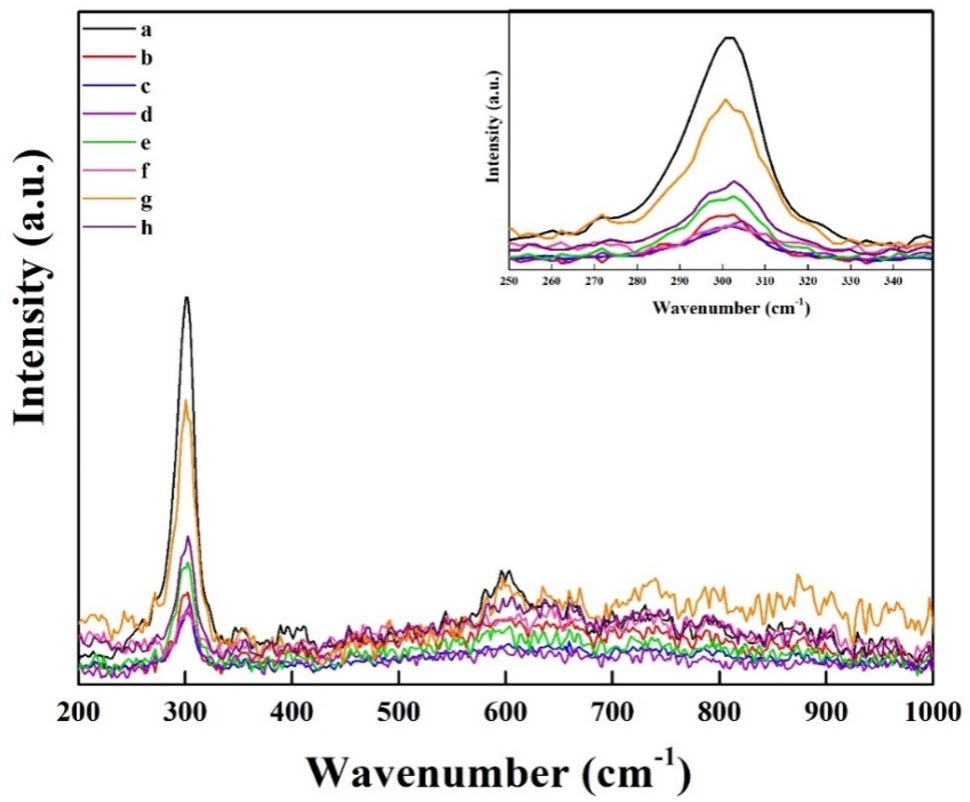
Raman spectra of optimum cases of CdS film with and without additives at: (**a**) Basic CdS, (**b**) CdS + Ag, (**c**) CdS + MPA, (**d**) CdS + salvia dye, (**e**) CdS + Ag + MPA, (**f**) CdS/Ag/salvia dye, (**g**) CdS + MPA + salvia dye, (**h**) CdS + Ag + MPA + salvia dye.

Vibrational spectroscopy is well known to be a very effective method for the estimation of the crystal phase. Raman spectroscopy offers an easy and non-destructive method for studying the crystalline structure and homogeneity of a semiconductor. The identification of lattice defects, dopant concentrations, and crystal orientations of materials is additionally performed out^42^. The identification of the peak location and width of the Raman spectrum offers valuable information regarding the crystalline properties of the film^43^. The Raman spectra of CdS films exhibit two different peaks, which may be attributed to the primary and secondary longitudinal optical phonon modes, during the chemical deposition process. The prominent and less prominent first and second longitudinal oscillation (1LO and 2LO) peaks were used to indicate the fundamental and overtone modes, correspondingly^44^. The results showed that after applying additives, 1LO peaks changed to lower values, and 2LO almost showed noise peaks, which could be attributed to element structure, crystallite size variance, mechanical stress in the interface, as well as an impurity within the film, Fig. 10.

It can be found in the inset of Fig. 10 that the 1LO phonon for cubic and hexagonal CdS structures indicates a small change from (301 to 305) cm^−1^, approving that there is no noticeable shift with the introduction of additives in the peak position. The diminished size of the CdS film is the likely cause of the decreased intensity of the lower 1LO peak value, which can be due to lattice softening^45^. The highest Raman line (LO) is attributed to sample (a), that is due to the sample quantity detected by Raman, which consider a good indication that the thin CdS nanostructured film formed by CBD is crystalline. More layers contribute, in other words, to fewer Raman improvements. Because it is induced by the transfer of charge between molecules and CdS. This is consistent with XRD outcomes. That is seen by modified lattice parameters, which will be responsible for lowering the frequencies of phonons. The findings presented in Sample (b) suggest that doping of longitudinal optical modes may result in downshifts, which could be attributed to reduced vibration frequencies resulting from the heightened density and diminished particle size of the doped nanocomposite.

This observation confirms the impact of the optical phonon mode on the surface. The reduction in particle size can be assigned to the breakdown of phonon momentum^46^. Furthermore, the introduction of Ag^2+^ doping results in lattice softening due to the replacement of Cd^2+^ positions by Ag^2+^, causing an overall downward shift of Raman modes towards lower phonon energies. That can be very clear in all cases that Ag^2+^ has been included. Sample (c) illustrates low intensity, which may be due to the effect of some existing unreacted sulphur atoms found on the surface. This is matching with X-ray diffraction, which has shown that annealing has activated the thiol group in MPA that has not to react between the CdS layers to form S_8_. In the case of samples (c, d, and f), the Raman spectra in the range from (270 to 330) cm^−1^ shows three related vibrational bands, depending on the content that has been included. The hexagonal level at low frequencies is commonly regarded as the regular peaks got through de-convolution using Lorentzian curves. These peaks are utilized for estimating the frequency of lattice modes^47^. The deposition of CdS film with a single additive resulted in an increase in FWHM. However, the addition of mixing additives, specifically samples e, g, and h, led to a lower drop in FWHM. This could be attributed to the presence of structural imperfections, such as voids, which were observed in the FESEM result. An increase in the region below the Raman peak was observed in the surroundings of bi-mixing. The reduction of the area below the Raman peak may be caused by lattice disturbance occurring during individual mixing events, as well as the influence of size on vibrational characteristics in small crystals^48^. In addition, a small rise in FWHM may be related to a slight degradation in the crystallinity of the CdS nanocrystals due to the introduction of both the Salvia dye and the MPA in the Cd sublattice into the sample (g). Both two have a smaller influence on the CdS structure relative to one of them alone. Thus, the second higher peak is attributed to the sample (g). Upon combining all the samples, the LO mode undergoes a slight shift due to the incorporation of all the additives. An insignificant reduction in Full Width at Half Maximum (FWHM) could potentially be explained by a slight enhancement in the crystalline structure of CdS. In this particular scenario, it is possible that the band has been assigned to undergo multi-phonon scattering. The presence of acoustic phonons in the scattering process results in the formation of a weak band, while the low-energy mode of the shoulder can be tracked back to the surface optical phonon mode^49^. The aforementioned factors together contribute to the observation that CdS displays a crystalline structural characteristic featuring a limited number of localized defects.

### CZTS solar cell fabrication and performance

In order to investigate the impact of doping and sensitization on the performance of CZTS solar cells, an experimental investigation was conducted whereby devices with different CdS buffer layers were fabricated. The selection of optimized cases has been approved according to their examined characterization on soda lime. Thus, it can be indicated three main categories according to optoelectronic, structure, morphology, and vibrational properties. First category that contained the highest results which is CdS + MPA, CdS + Ag + MPA, and CdS + mix all. Second category consists of moderate results, as CdS, CdS + Ag, and CdS + salvia dye. While the last category is for both CdS + Ag + salvia dye, and CdS + MPA + salvia dye that have low optoelectronic properties compared with other samples. Thus, the first and second categories have been chosen as six buffer layers used to fabricate CZTS solar cells.

In terms of fabrication, it cannot have compared the impact of a single layer alone with the same layer after being deposited on the top of the absorber layer (for example CZTS in this work). Through the observation, it can see clearly how the effect of CdS + MPA is very important as a single layer, but after deposition on the CZTS, it is harming the cell and led to damage it. While through using another natural organic material (Salvia dye) showed high coverage with good thickness. That is possibly due to as a natural dye, it is not contained a strong complex as that is found in MPA, so with-it simple complexes that dissolved very easily in the reaction to synthesis CdS in the presence of CZTS, it produced good CdS/CZTS compared with all samples. Both CdS + Ag, and CdS + Ag + MPA have shown less thickness and black dark color. As mentioned earlier, Ag when it is mixed with MPA, led to disturbing the reaction, but in terms of fabrication, this led to reduce the effect of MPA and prevents the damaging. Besides, basic CdS showed good coverage. Partial damage has been notifying also for (CdS + mix all) sample, probably in this case the influence of each additive has shown an effect on each other.

Additional insight into the impact of the different additives on the CZTS device operation was obtained by I–V measurement. The I–V curve represented the relationship between the current density (J) with voltage (V) which is different from the short-circuit current (J_SC_) and open-circuit voltage (V_OC_). The short-circuit current is applicable to the electric current that flows through a solar cell when the voltage across it is at zero. On the other hand, the open-circuit voltage (V_OC_) refers to the highest voltage that a solar cell can generate, which happens when there is no current flowing through it. Also, the current (I) is the short circuit current density (J_SC_) times the cell area:

2 $$I={J}_{SC} \cdot Area$$

**Figure 11:**
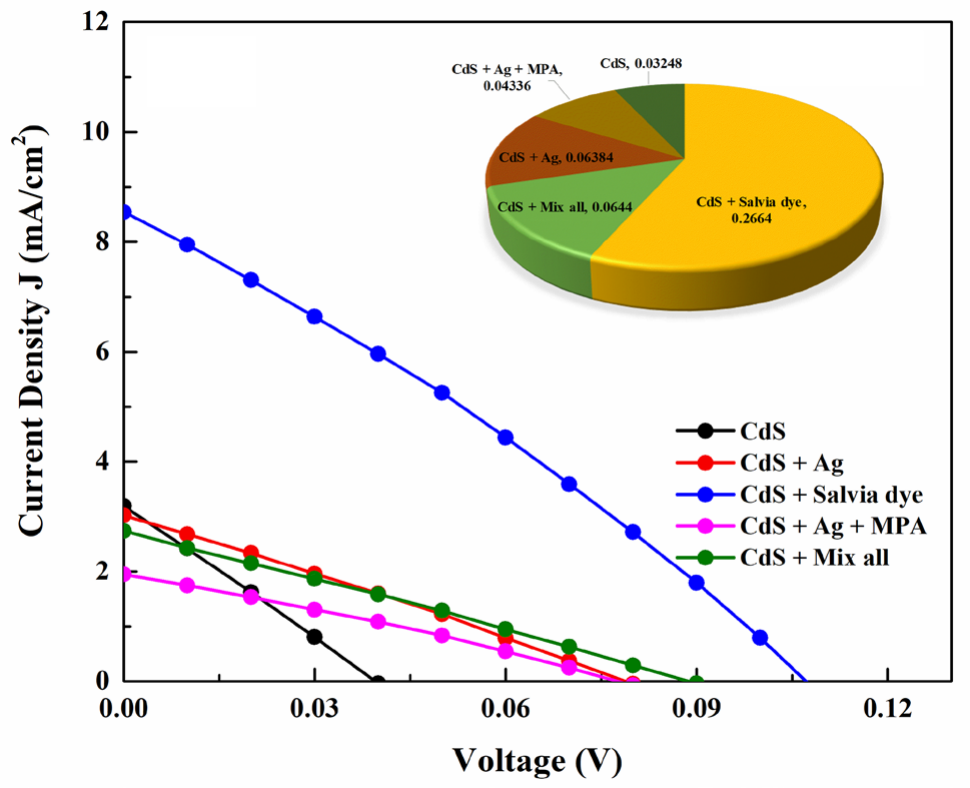
I–V curve of the CZTS device fabricated.

The different values of J_SC_ and V_OC_ have been shown in Table 3; Solar cell output parameters are calculated from the I–V curve. The calculation of I–V was achieved under A.M. 1.5 solar illumination, Fig. 11.

**Table 3:** Solar cell parameters from the I–V curve.

Samples	J_sc_ (mAcm^−2^)	V_oc_ (V)	FF	η (%)
CdS	3.196	0.039	0.256	0.032
CdS + Ag	3.024	0.078	0.267	0.063
CdS + salvia dye	8.544	0.107	0.290	0.266
CdS + Ag + MPA	1.944	0.078	0.285	0.043
CdS + mix all	2.744	0.089	0.263	0.064

As a whole, it was shown that J_SC_ is very sensitive to carrier mobility and carrier concentration in the CdS buffer layer for the absorber layer. Accordingly, the first category should be recorded as the highest value, but after deposition on the CZTS, CdS + MPA harm the cell and led to damage it. This is could be due to the effect of MPA as a strong organic acid within CZTS via CdS/CBD. On the other hand, the sample that included Salvia dye showed a higher value, this can be predicated as the presence of carboxyl groups on the dye expected to contribute to higher J_SC_ values, thereby increasing electron diffusion from the dye molecules to (CdS/CZTS)^26^.

It is anticipated that the V_OC_ would similarly exhibit an increase in both the CdS + Salvia dye and CdS + Mix all samples. This may be attributed to the fact that Salvia dye has the potential to function as an electron transport medium, resulting in higher currents and therefore leading to enhanced power conversion efficiency^50^. The basic CdS exhibited the lowest amount of V_OC_, which may be ascribed to the initiation of the recombination process at the interface between the buffer and absorber layers^51^.

The dropped FF values recorded in this study were ascribed to a reduction in electron injection efficiency on the CdS/CZTS interface. The rise in the value of η observed between the two samples, namely CdS + Salvia dye and CdS + Mix all, may be attributed to the existence of secondary phases resulting from the influence of organic complexes, that led to the high bandgap of these phases, which could improve directly the performance of the solar cells. Another slight improvement has been carried out for CdS + Ag and CdS + Ag + MPA. While the lowest η has been recorded to basic CdS. The maximum conversion efficiency recorded for a CZTS thin-film solar cell in this work is 0.266% by using a CdS + Salvia dye sample, Table 3.

**Figure 12:**
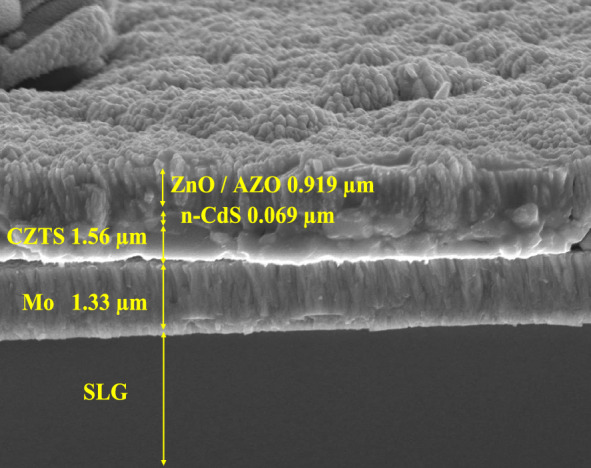
FESEM cross-section for CZTS + Salvia dye full device.

In the contrary, the increased effectiveness of the Salvia dye was a result of its high current density. The solar device's strong absorbance and low electrical resistance led to its high current density values, which translated to a high device performance. Figure 12 shows the cross-section for CZTS full device while using Salvia dye. Each layer's thickness was determined using the FESEM image to be 1.33 μm for Mo, 1.56 μm for CZTS, 0.06 μm for n-CdS, and 0.19 μm for ZnO/AZO. It was challenging to distinguish apart the different buffer and window layers (n-CdS/ZnO/AZO). The CZTS grains appear to be strongly affixed to the Mo layer and quite compact. Sputtering is often used for producing the Mo back contact layer on SLG substrates, and this layer frequently has a columnar grain shape. In the solar cell, the beneficial contact between the surface of the CZTS absorber layer and the Mo back contact helps to reduce minority carrier recombination and provides a current channel for minority carriers to reach the buffer layer n-type CdS.

## Conclusions

An interesting and effective hybrid sensitization approach has evolved, which combines the one-step linker of the assisted chemical bath deposition technique with conventional CBD doping. The use of this approach has led to the fabrication of improved CdS thin films. Using a basic CdS-CBD in the reaction solution, the causing films possess a compact microstructure layer. The layer is developed during the reaction via adding (Ag, MPA, and Salvia dye) it reaches the best characterisation by mixing each one with another. The energy band of films exhibited an increase in peak intensity upon the introduction of additives during the deposition reaction. This observation suggests a reduction in the number of impurities, defect centres, and surface state recombination reactions. Based on electron diffraction patterns, the crystal structure of the particles was determined, and evidence was given for the bonding of thiolate ions to cadmium ions on the surface of the particles. Uniform, granular, continuous, smooth surfaces and fewer pinholes were observed for most of the samples. The optimal morphological characterisation of the films reveals very homogeneously distributed spherical grains in the (CdS + MPA) sample. The hexagonal structure of CdS thin films was verified through Raman spectroscopy, wherein the identification of the first longitudinal optical phonon mode occurred at a Raman shift of 302 cm^−1^, while the second LO was observed at 605 cm^−1^. All the films related to CdS modified method lead to the conclusion that CdS with mixing has a crystalline structure quality with a few local defects. Finally, rudimentary CdS/CZTS solar cell structures have been fabricated to elucidate the applicability of these improved CdS films. CZTS thin film solar cell with the maximum conversion performance obtained in this study refers to the (CdS + Salvia dye) sample with 0.266%, suggesting the possible usage of CdS films with controllable doping.

## Data Availability

The datasets used and/or analysed during the current study available from the corresponding author on reasonable request.
